# Oral symptoms and oral health-related quality of life of individuals with x-linked hypophosphatemia

**DOI:** 10.1186/s13005-019-0192-x

**Published:** 2019-03-23

**Authors:** Marcel Hanisch, Lauren Bohner, Martin M. I. Sabandal, Johannes Kleinheinz, Susanne Jung

**Affiliations:** 1 Department of Cranio-Maxillofacial Surgery, Research Unit Rare Diseases with Orofacial Manifestations (RDOM), University Hospital Münster, Albert-Schweitzer-Campus 1, Gebäude W 30, 48149 Münster, Germany; 2 Central Interdisciplinary Ambulance in the School of Dentistry, University of Münster, Albert-Schweitzer-Campus 1, Building W30, Waldeyerstrasse 30, 48149 Münster, Germany

**Keywords:** Rare diseases, Oral health related quality of life, OHRQoL, XLH, X-linked hypophosphatemia, OHIP-14

## Abstract

**Background:**

The primary purpose of this study was to collect data on the oral health-related quality of life (OHRQoL) of individuals with x-linked hypophosphatemia (XLH). It was also designed to gather information on the period of diagnosis, oral symptoms, orthodontic therapy, and satisfaction with dental care and the healthcare system.

**Methods:**

A questionnaire was developed to evaluate the OHRQoL consisting of open-ended questions and the standardised German version of the Oral Health Impact Profile-14 (OHIP-14).

**Results:**

The questionnaires from 43 participants were analysed, including 32 females (74.41%) and 11 males (25.59%). The mean OHIP-14 total score for the combined genders was 10.30 points (range: 0–37 points). For the combined genders, the mean period of time that elapsed between the first signs of the illness and the diagnosis was 5.52 years (range: 0–49 years). In total, 77.50% of the participants described oral symptoms, such as tooth mineralisation defects (*n* = 26), abscess or fistula formation (*n* = 21), dysgnathia (*n* = 9) and temporomandibular dysfunction (n = 2). The correlation between the participants’ satisfaction with the healthcare system and the OHIP-14 values was weak (− 0.21), and it was not statistically significant (*p* = 0.199).

**Conclusions:**

The majority of the study participants reported oral involvement in the context of XLH, especially dental hard tissue mineralisation disorders, abscess formation and fistula formation. Those individuals affected by XLH with oral manifestations exhibited a tendency toward a worse OHRQoL than those without oral symptoms. In Germany, the OHIP-14 scores for these XLH patients were worse than those values that were obtained from the general population.

**Electronic supplementary material:**

The online version of this article (10.1186/s13005-019-0192-x) contains supplementary material, which is available to authorized users.

## Background

In the European Union, a disease is considered to be rare if it affects less than one in 2000 people [1]. Rare diseases have been an increased focus of public awareness in the European Union since 2009, when the Council of the European Union asked the Member States to develop plans and strategies in this regard [2]. X-linked hypophosphatemia (XLH) is the most common form of vitamin D-resistant rickets [3], and with a prevalence of 4.8 cases per 100,000 individuals [4], it is considered to be a rare disease. XLH is caused by a mutation in the x-linked phosphate-regulating neutral endopeptidase (PHEX) gene found on chromosome Xp22 [5]. Mutations in the PHEX gene are the main reasons for the emergence of this vitamin D-resistant form of rickets. They give rise to dysregulation in the form of an increase in the expression of fibroblast growth factor 23, which in turn results in phosphaturia, hypophosphatemia and the impaired activation of 25-hydroxyvitamin D to 1,25-dihydroxyvitamin D [6]. Although XLH can vary in terms of its clinical manifestations, it is usually characterised by bone deformities, a small body size and dental anomalies. This is due to reduced renal phosphate reabsorption [7], which results in hypophosphatemia and reduced bone and tooth mineralization [8].

### Dental abnormalities phosphate-regulating endopeptidase gene

The dental findings in XLH cases are diverse, and the exact pathogenesis remains unclear [9]. The most common oral findings are recurrent abscesses and/or fistulas in caries-free teeth in both the primary and permanent dentition phases. Other symptoms include dentitio tarda (late secondary dentition) and radiographically visible, severely enlarged pulp chambers [9]. From a histological perspective, poorly mineralised dentin dysplasia has been found in both the deciduous and permanent teeth [10–12]. The dentin mineralisation defects appear as characteristically large interglobular spaces resulting from the lack of calcospherite fusion in the circumpulpal region during the mineralization process [13], with large crevices or lacunae within the dentin. The odontoblast function is not restricted, but rather, hypophosphatemia is the cause of the dysplastic and sparsely mineralised circumpulpal dentin, with large areas of interglobular dentin [14]. Enamel hypoplasia is significantly more common in the incisors, canines and first permanent molars in XLH cases than in the premolars and other molars, but overall, this does not appear to be a predominant symptom [15].

**Fig. 1:**
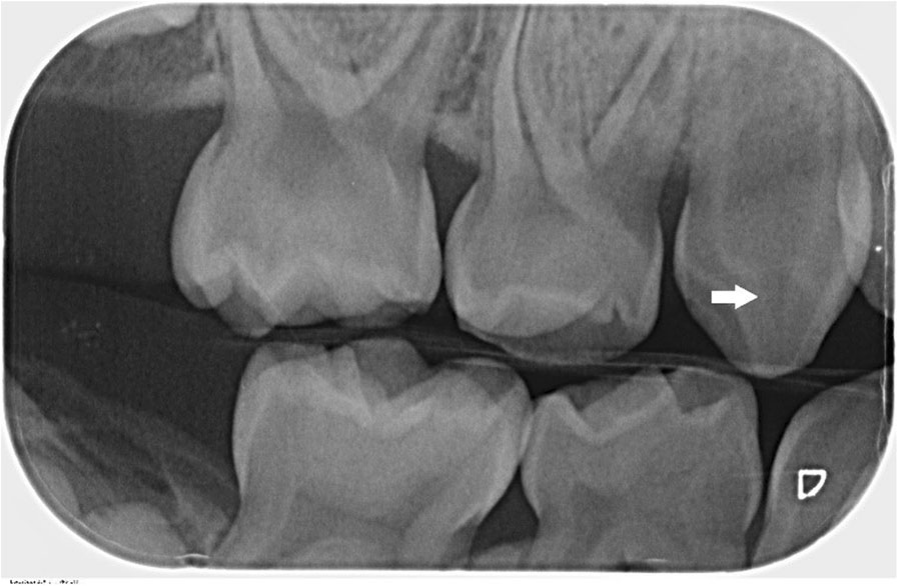
Enlarged pulp horn extending to the enamel cement boundary (arrow) in deciduous tooth 53 in a five-year-old boy with x-linked hypophosphatemia

**Fig. 2:**
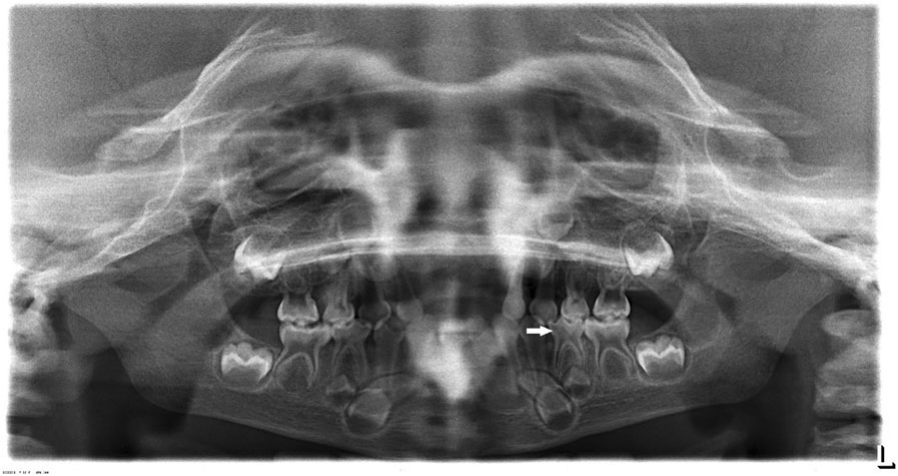
Panoramic image of a 5-year old boy patient with x-linked hypophosphatemia with pronounced pulp horns (arrow)

Odontogenic abscesses in caries-free teeth are often observed in those affected by XLH [16, 17]. In general, XLH calls for a high degree of endodontic therapy due to apical periodontitis [18]. Such apical periodontitis (and the associated endodontic treatments) can be caused by multiple alterations in the dentin and enamel. Radiographically, large pulp chambers can be found, which reduce the thickness of the dentin and enamel. Histopathologically, the dentin shows various structural abnormalities caused the lack of calcospherite confluence, resulting in large interglobular spaces and unmineralized dentin. Conversely, the enamel is regularly formed with long cracks [9]. Because the teeth are constantly exposed to masticatory forces, the altered dentin can be exposed, resulting in the bacterial contamination of the dental tissues (Figs. 1 and 2) [9, 19, 20]. The increased need for endodontic treatment is correlated with the increased age of the population affected by XLH, which implies a link between attrition/abrasion and occurrence of apical periodontitis [21]. The pathogenesis of x-linked hypophosphatemia is shown in Additional file 1.

When endodontic treatment is required, the risk of reinfection increases due to the altered dentin structure and large interglobular spaces [9]. The bacterial contamination due to the dental structure alterations differs from that due to decay in such a way that, when performing endodontic treatment, there is an increased risk of further contamination due to the structural defects [9].

To date, there have been only a few studies of the oral health-related quality of life (OHRQoL) of individuals with rare diseases [22]. Among these studies, the participants tended to report a lower OHRQoL; however, the authors were unable to find any reports about the OHRQoL of individuals affected by XLH.

The Oral Health Impact Profile-14 (OHIP-14) questionnaire, which measures the incidence of 14 different functional and psychosocial influences on the OHRQoL [23], has been proven to be a useful tool for assessing the OHRQoL. The primary purpose of this study was to collect data on the OHRQoL of people with XLH. It was also designed to gather information regarding the diagnosis period, oral symptoms, orthodontic therapy, satisfaction with dental care and satisfaction with the healthcare system.

## Methods

### Study design

This study was designed as an anonymous epidemiological survey among individuals with XLH in order to evaluate their OHRQoL. A questionnaire was developed for this purpose consisting of open-ended questions and the standardised German version of the OHIP-14 questionnaire [24]. Each of the 14 questions of the OHIP-14 questionnaire were assigned standardised numerical values as follows: 0 = never, 1 = rarely, 2 = now and then, 3 = often and 4 = very frequently. The potential overall minimum and maximum scores were 0 and 56 points, respectively. The higher the numerical score, the worse the participant’s OHRQoL, and the questions were related to the participants’ experiences during the preceding month. The questionnaires were sent digitally to the XLH self-help group (Phosphatdiabetes e.V.) registered with the umbrella organisation of self-help groups for chronic rare diseases in the Federal Republic of Germany, the Alliance for Chronic Rare Diseases (Allianz Chronisch Seltener Erkrankungen e.V.).

This study was approved by the ethics committees of the Chamber of Physicians of Westfalen-Lippe (Ärztekammer Westfalen-Lippe) and the University of Münster in Germany (Ref. No. 2016–006-f-S).

### Participants

Anyone living in Germany, with a minimum age of 16 years old, who was affected by XLH was eligible to participate in this study. Because the questionnaires were only sent to the members of a self-help group, the range of participants was largely limited. According to the information provided by the self-help group, it included 90 members at the time of this study.

### Data source

Along with the age, gender and disease status, the questionnaire asked the participants about their oral symptoms, diagnosis age, time period between the first disease signs and the diagnosis, satisfaction with their dental care and satisfaction with the German healthcare system. Additionally, the OHIP-14 values were calculated. The information provided by the patients about their oral symptoms was translated into the relevant medical terminology; for example, if a patient reported that their ‘jaw was too small’, this was recorded as ‘dysgnathia’.

### Statistical analysis

The statistical analysis was performed using IBM SPSS Statistics for Windows version 22.0 (IBM Corp., Armonk, NY, USA). The data were described based on the mean and percentage values. The data’s adherence to the normality curve was assessed using the Shapiro-Wilk’s test. Additionally, a chi-squared test was performed to assess the relationships between the categorical groups, while the Student’s t-test was used to compare the normally distributed means. Conversely, the nonparametric Kolmogorov-Smirnov z-test was used when the data did not adhere to a normal curve. The correlations between the variables were assessed using Pearson’s correlation test.

## Results

### Participants

**Fig. 3:**
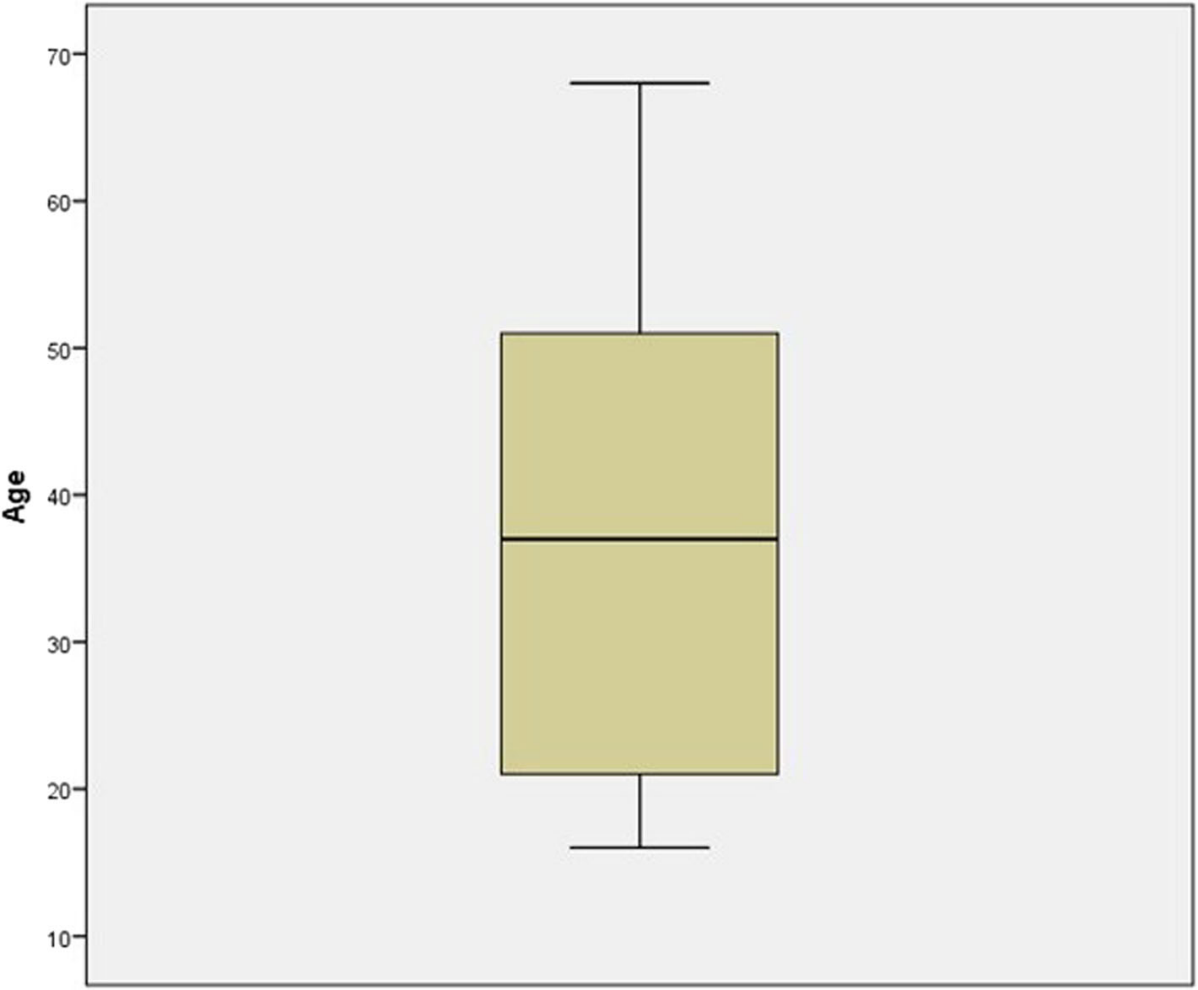
Age and gender from the participants

In total, 43 questionnaires were analysed from 32 female (74.41%) and 11 male (25.59%) participants. The mean age of the combined genders was 37.30 years old (range: 16–68 years old). In the female participants, the mean age was 40.34 years old (range: 16–68 years old), while the mean age of the male participants was 28.45 years old (range: 16–56 years old). The descriptive data are shown in Fig. 3.

### Diagnosis age and diagnosis period

The mean XLH diagnosis age of the combined genders was 9.82 years old (range: 0–49 years old). The average diagnosis age of the female participants was 9.16 years old (range: 0–40 years old), while the average diagnosis age of the male participants was 11.69 years old (range: 0.17 months to 49 years). No statistically significant differences were found between these groups (*p* = 0.862).

When considering the combined genders, the mean period of time between the first signs of illness and the diagnosis was 5.52 years (range: 0–49 years). For the female participants, the elapsed time was 5.06 years (range: 0–32 years), and for the male participants, the elapsed time was 6.81 years (range: 0–49 years). However, the difference between the genders was not statistically significant (*p* = 0.957).

### Oral symptoms

Forty participants responded to the questions about their oral symptoms, of which 31 (77.50%) described some oral symptoms (23 women and 8 men) and nine (22.50%) reported no oral symptoms related to their disease (7 women and 2 men). The appearance of oral manifestations was statistically similar between the male and female participants (*p* = 0.295).

The following oral symptoms were reported: tooth mineralisation defects (*n* = 26), abscess or fistula formation (*n* = 21), dysgnathia (*n* = 9) and temporomandibular dysfunction (n = 2).

### OHIP-14 values

The total OHIP-14 score mean for the combined genders was 10.30 points (range: 0–37 points). For the female participants, the mean score was 10.25 points (range: 0–37 points), and for the male participants, the mean score was 10.45 points (range: 0–28 points). The difference between the OHIP-14 scores of the male and female participants was not statistically significant (*p* = 0.944).

**Fig. 4:**
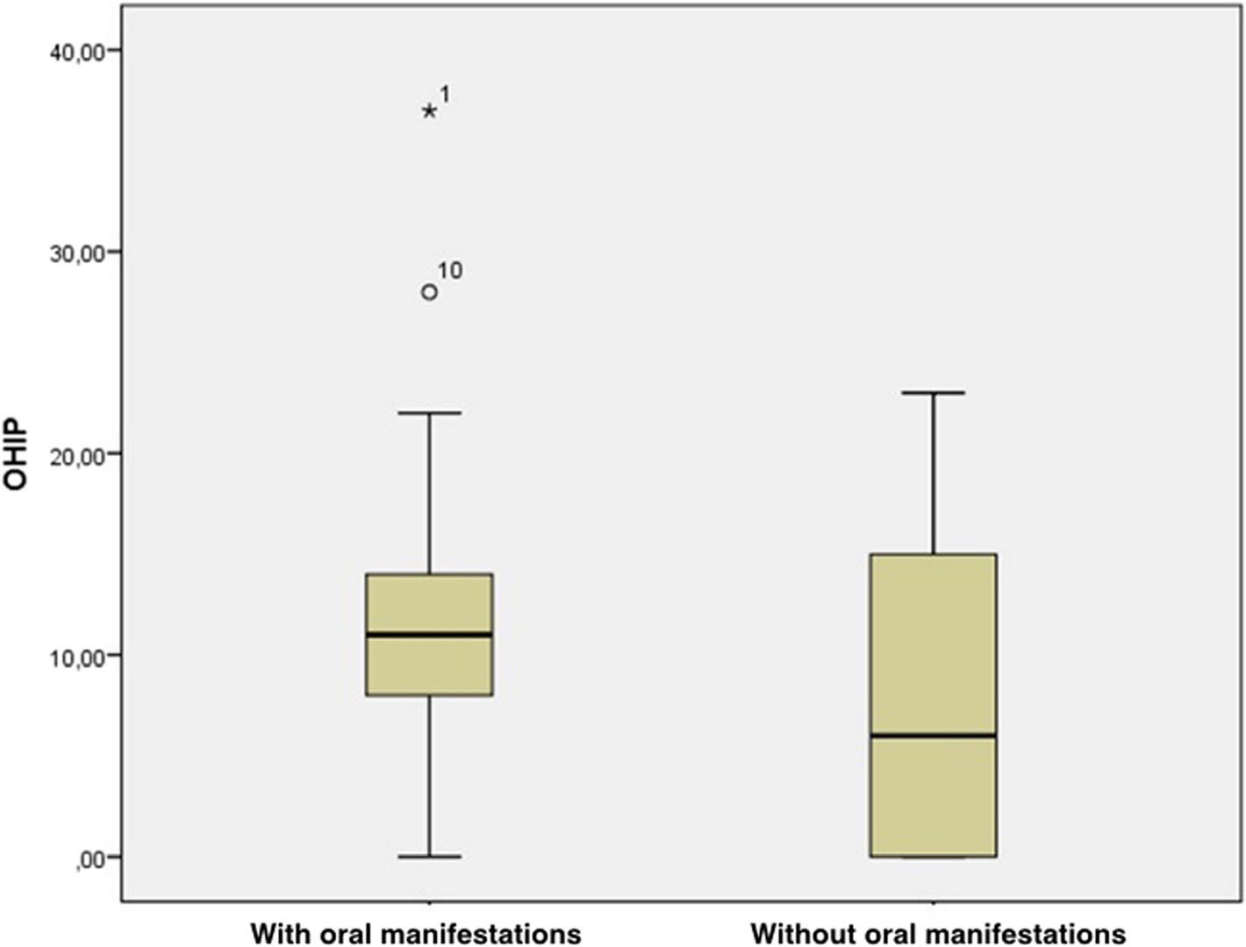
Box-plot showing OHIP values (mean and interquartile range) for patients with and without oral manifestations. Symbols (star and circle) indicate outliers of the study sample

In the group that did not report any oral symptoms, the mean OHIP-14 score was 7.91 points (range: 0–23 points), while the group with oral symptoms had an mean score of 11.23 points (range: 0–37 points). The difference between the scores of these two groups was not statistically significant (Table 1, Fig. 4).

**Table 1:** OHIP values between patients with and without oral manifestations (OM)

	Mean (SD)	Median	95% Confidence Interval	*p*-value
With OM	12.18 (8.76)	11.00	(8.29;16.06)	0.295
Without OM	7.91 (8.12)	6.00	(2.75;13.08)

### Satisfaction with perceived dental care quality

A total of 37 participants were satisfied with their perceived dental care quality, and this group had an mean OHIP-14 score of 10.37 points (range: 0–37 points). Five of the participants were unhappy with their dentists, and their mean OHIP-14 score was 9.80 points (range: 5–15 points). No correlation was found between the participants’ dental satisfaction and the OHIP-14 values (*p* = 0.619).

### Healthcare system satisfaction

A total of 8 participants were satisfied with the dental care aspects of the German healthcare system, and this group had an mean OHIP-14 score of 7.37 points (range: 0–15 points). Thirty of the participants were unsatisfied with the German healthcare system and they had a mean OHIP-14 score of 10.97 points (range: 0–37 points). The correlation between the participants’ satisfaction with the healthcare system and their OHIP values was weak (− 0.21), and it was not statistically significant (*p* = 0.199). The results are shown in Table 2.

**Table 2:** Age and sex of participants, age of diagnosis, time interval from onset of the first symptoms to diagnosis, possible oral involvement, satisfaction with perceived quality of care by their dentist, satisfaction with health system and determined OHIP values. (n/s = not specified; F = female; M = male)

Gender, Age	Diagnosis age	Period, which elapsed from the first signs to diagnosis (in years)	Oral symptoms	Satisfied with perceived quality of care by their dentist	Satisfied with health system	OHIP-14 Score
F, 60	27.5	25	n/s	No	Yes	15
F, 48	12	12	disturbance of mineralisation, abscess/fistula	Yes	No	37
F, 46	1.5	0.75	disturbance of mineralisation, abscess/fistula	n/s	No	4
F, 23	1.25	0.5	disturbance of mineralisation, abscess/fistula	Yes	Yes	14
M, 20	0.17	0	disturbance of mineralisation, abscess/fistula, dysgnathia	Yes	No	13
M, 17	4.5	0	disturbance of mineralisation, abscess/fistula	Yes	No	10
F, 46	4	3	disturbance of mineralisation, abscess/fistula	Yes	No	22
F, 51	15	0.15	n/s	Yes	Yes	5
M, 51	49	49	n/s	Yes	No	7
F, 51	6	4	disturbance of mineralisation, abscess/fistula	Yes	No	2
F, 42	n/s	n/s	disturbance of mineralisation, abscess/fistula	Yes	No	12
F, 16	11	6	dysgnathia	No	No	11
M, 17	17	10	hypodontia	Yes	No	28
F, 44	2	2	disturbance of mineralisation, abscess/fistula	Yes	No	22
F, 49	38	32	disturbance of mineralisation, abscess/fistula	Yes	No	13
F, 17	1.5	0.33	No	Yes	Yes	0
M, 36	2	0.5	disturbance of mineralisation, abscess/fistula, dysgnathia	Yes	No	3
F, 53	6	5	disturbance of mineralisation, abscess/fistula	Yes	No	8
F, 22	0.25	0	disturbance of mineralisation, abscess/fistula	Yes	No	16
F, 62	27	24	dysgnathia	No	Yes	12
M, 20	4.5	2.5	disturbance of mineralisation, abscess/fistula	Yes	No	8
F, 26	3	0.1	disturbance of mineralisation, abscess/fistula	Yes	n/s	4
F, 36	1.5	0.5	disturbance of mineralisation, dysgnathia	Yes	No	11
M, 48	45	9	No	Yes	No	23
F, 24	5	4	disturbance of mineralisation, abscess/fistula	Yes	No	0
F, 29	2.5	1	disturbance of mineralisation, TMD	Yes	Yes	8
F, 48	15	1.5	disturbance of mineralisation, abscess/fistula	Yes	No	10
F, 68	12	5.5	No	Yes	No	16
F, 57	4	3	No	Yes	No	15
F, 37	0	0	No	Yes	No	1
F, 17	0	0	No	Yes	No	0
F, 33	0	0	No	Yes	No	0
M, 16	2.5	2	disturbance of mineralisation, abscess/fistula	Yes	n/s	0
F, 52	40	n/s	dysgnathia	Yes	n/s	6
M, 16	0.6	0.6	No	Yes	No	13
F, 56	2	2	dysgnathia, TMD	Yes	No	8
M, 56	3	1	disturbance of mineralisation, abscess/fistula	No	n/s	5
F, 16	6	1.5	No	Yes	Yes	0
F, 47	4	3	disturbance of mineralisation, dysgnathia	Yes	No	18
F, 28	19	6	disturbance of mineralisation, abscess/fistula	No	No	6
F, 53	15	13	disturbance of mineralisation, abscess/fistula	Yes	No	13
M, 16	0.33	0.33	disturbance of mineralisation, abscess/fistula	Yes	Yes	5
F, 34	2	1	disturbance of mineralisation	Yes	n/s	19

## Discussion

The overwhelming majority of the study participants were females (74.41%). However, this should not be taken as an indication of a higher XLH prevalence among women. Actually, there is actually no difference in the XLH prevalence between the genders [25].

On average, a rare disease is present for 7 years before it is correctly diagnosed [26], and the oral manifestations can serve as an important indication of the underlying disease [27]. For the combined genders, the mean time between the participants’ first symptoms and their XLH diagnosis was 5.52 years. In this case, the time period was below the 7 year average that has been described as typical for rare diseases [26]. However, the male participants tended to be diagnosed later (6.81 years) than the females (5.06 years). Overall, the individuals with XLH appeared to benefit from an earlier diagnosis than that which is usually reported for rare diseases. Perhaps the oral manifestations associated with XLH led to an earlier diagnosis of this rare disease, and this has been ascertained by other studies [22]. A majority of the participants (77.50%) reported oral symptoms in connection with XLH. This group experienced hard tissue mineralisation (*n* = 26) and abscess or fistula formation (*n* = 21). These symptoms have also been described in the literature, and they can affect both the permanent and deciduous teeth [9].

Although the symptoms described by the participants were not clinically verified by the authors, the results appeared to confirm those of previous reports of abscess or fistula formation on caries-free and trauma-free teeth [9, 19]. However, not all of the study participants with XLH appeared to be affected by the clinically apparent dental hard tissue symptoms. At least 22.50% of the study participants reported no oral manifestations at all, which may point to the existence of varying XLH subtypes: some with clinically relevant dental hard tissue involvement and others without those symptoms. This must be investigated in the future with epidemiological and molecular genetic studies.

While craniosynostosis has previously been described as a possible craniofacial manifestation of XLH [28], there are no references to dysgnathia in the literature. However, with 88.37% of the respondents reporting that they had previously undergone orthodontic treatment, potential skeletal dysgnathia in connection with XLH merits further investigation. In the group questioned here, 9 participants mentioned dysgnathia, although these cases were not clinically evaluated by the authors.

In general, the literature is contradictory as to whether men are more likely to be affected by more severe forms of XLH or whether there are no gender-specific differences [18]. For the combined genders, the mean XLH OHRQoL was 10.30 points on the OHIP-14 scale, and the values obtained for the separate genders (10.25 points for the males vs. 10.45 points for the females) did not differ. Unfortunately, comparative values from other OHRQoL studies of individuals with XLH were not available, or the authors were unable to identify them. However, there were some comparative values available from a representative study of the German general population, for whom a mean OHIP-14 value of 4.09 points was calculated [29]. Standard values can be used to interpret the impaired OHRQoL level of specific individuals and groups of individuals in relation to their impaired OHRQoL degree among the general population [30]. This demonstrated that the individuals with XLH in Germany exhibited a worse OHRQoL when compared to the general population.

The OHIP-14 score for the group without oral symptoms was 7.91 points, while the group reporting oral symptoms showed a higher average score of 11.23 points. Although it was not statistically significant, there was a higher discrepancy among the OHIP-14 values of the participants without oral manifestations, while those who reported oral symptoms had a tendency to report greater OHIP-14 values. This allowed us to conclude that the individuals with XLH and oral involvement exhibited worse OHRQoL values than those with no oral symptoms.

Because XLH patients must maintain good oral hygiene, with regular dental check-ups and prophylactic measures (such as fissure sealing) [31], there is a high necessity for trusting relationships with their dentists. Fortunately, 88.10% of the respondents reported that they were satisfied with their perceived dental care quality. There seemed to be no issues related to dental care in the XLH patients in Germany. However, 78.95% of the participants were dissatisfied with the support of the German healthcare system in relation to their dental care. Among this group, the OHIP-14 scores were worse (10.97) than the scores among those who were satisfied with the healthcare system (7.37).

At this point, we can only speculate about the reasons why people with XLH are dissatisfied with the dental care provided by the German healthcare system. One possible reason could be that the prophylactic measures, such as partial fissure sealing or general teeth cleaning, must be paid for on a completely private basis, although these are necessary as basic preventative measure in XLH cases. In this regard, and based on the German Social Code V, § 28 [32], which states that ‘… Dental treatment includes any activities by the dentist that are adequate and appropriate for the prevention, early detection and treatment of dental, oral and jaw diseases according to the accepted state of the art …’ [32], it may be necessary for statutory health insurance schemes to reimburse prophylactic measures aimed at preventing specific complications in connection with XLH.

The main limitation of this study was that the study population was restricted to the participants of a self-help group in Germany, of which all 90 members were diagnosed with this rare condition. Thus, it is questionable whether the present findings may be extrapolated to the general population of people affected by XLH. In addition, the chosen study design did not allow for the authors’ clinical examination of the oral conditions. Further studies are required to investigate how specific oral manifestations, such as large pulp chambers and periodontal manifestations, can influence the OHRQoL of people affected by XLH.

## Conclusions

The majority of the study participants reported oral involvement in the context of XLH, especially hard tooth tissue mineralisation disorders, abscess formation and fistula formation. Moreover, the XLH patients with oral manifestations exhibited a tendency toward a worse OHRQoL than those without oral symptoms.

## Additional file

Additional file 1: Pathogenesis of x-linked hypophosphatemia (JPG 44 kb)

## Abbreviations

ACHSE: Alliance of Chronical rare Diseases
EU: European Union
NAMSE: National Plan of Action for People with Rare Diseases
OHIP: Oral Health Impact Profile
OHRQoL: Oral Health related quality of life
PHEX: Phosphate Regulating Endopeptidase Gene
XLH: X-linked Hypophosphatemia
