# Supplementary figures and images for: Oral symptoms and oral health-related quality of life of individuals with x-linked hypophosphatemia
Source: Head Face Med. 2019 Mar 23;15:8. doi: 10.1186/s13005-019-0192-x (PMC6431058; doi:10.1186/s13005-019-0192-x)

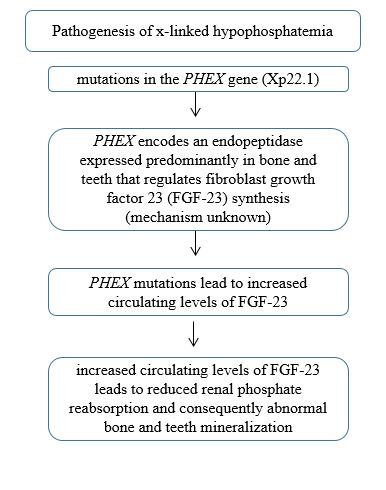

Supplement: Supplementary file 1 — Pathogenesis of x-linked hypophosphatemia (JPG 44 kb) [file 13005_2019_192_MOESM1_ESM.jpg]
